# Women in vision networks: narrowing the global gender gap in academia

**DOI:** 10.1177/25158414261451672

**Published:** 2026-06-02

**Authors:** Louise Terry, Bethany E. Higgins, Dillys A. D. Amega, Enyam K. A. Morny, Lindsay Rountree

**Affiliations:** School of Optometry and Vision Sciences, Cardiff University, Maindy Road, Cardiff CF24 4HQ, UK; Optometry and Visual Sciences, School of Health & Psychological Sciences, City St George’s, University of London, London, UK; Women in Vision UK, Liverpool, UK; School of Optometry and Vision Science, University of Cape Coast, Cape Coast, Ghana; Women in Vision Network, Cape Coast, Ghana; School of Optometry and Vision Science, University of Cape Coast, Cape Coast, Ghana; Women in Vision UK, Liverpool, UK; School of Pharmacy, Optometry and Medical Sciences, University of Bradford, Bradford, UK

**Keywords:** network, ophthalmology, vision science, women

Despite decades of progress towards gender equity in science, the persistent underrepresentation of women in healthcare leadership and academia remains a significant and systemic challenge. In the United Kingdom, 42% of ophthalmologists are women, but this drops to 36% at the consultant level.^1^ This trend is perhaps more striking at a global level, with an estimated 75% of leadership roles in healthcare worldwide held by men, despite comprising only ~30% of the healthcare workforce.^2,3^ Evidence shows the underrepresentation of women in healthcare leadership roles undermines healthcare systems, leading to less effective policies for women and children, diminished workforce morale, increased financial burdens and a loss of critical skills and experience.^4^

This trend is not limited to clinical spaces, with academic careers showing a similar pattern. In the United Kingdom, women make up 56% of the undergraduate population, but only 45% of academic jobs.^5^ This gap widens with increasing seniority; just 21% of professors are women. A 2023 report from the United Nations Educational, Scientific and Cultural Organization (UNESCO) on the representation of women in academia and higher education management positions showed that gender inequity in tertiary education was particularly striking in Sub-Saharan Africa, with 10 countries having <20% female representation in academic staff.^6^ This is echoed by Education Sub Saharan Africa (ESSA), who found that, despite women comprising 43% of students in tertiary education, women hold only 8% of professorships in Ghana, and 2.5% of vice-chancellor positions across Sub-Saharan Africa.^7^ It is important to note that gender often intersects with other structural barriers (e.g. socioeconomic background, care responsibilities), reinforcing the need for inclusive and context-sensitive support networks.

The importance of gender equity in academia is well recognised, with growing support from national and local governments and non-governmental organisations (NGOs) alike. In 2021, the British Council launched the ‘Gender Equality Partnerships’ scheme, aimed at addressing global gender challenges in further and higher education.^8^ In total, the scheme has supported 113 partnerships between the United Kingdom and 13 countries worldwide, addressing areas such as tackling subject segregation in science, technology, engineering and mathematics (STEM) subjects, establishing higher education institutions (HEIs) as safe spaces for women, and addressing women’s underrepresentation in higher education leadership. Targeting the latter, a partnership brought together researchers from Cardiff University in the United Kingdom, the University of Cape Coast (UCC) in Ghana and Women in Vision UK, with three main aims:

(1) Understanding the barriers and motivations for career choices in vision science, with respect to gender
(2) Launching a support network for promoting and empowering women aspiring to pursue academic careers in vision science
(3) Establishing a peer-mentoring scheme for members of the network

The project began with a scoping exercise to explore the academic landscape and gain a deeper understanding of the challenges that may influence career decisions, particularly in the pursuit of academic roles. It specifically examined how these motivations and barriers differ by gender. The School of Optometry and Vision Science at the UCC and the Department of Optometry and Visual Science at Kwame Nkrumah University of Science and Technology (KNUST; the two HEIs in Ghana currently offering an optometry programme) both took part in this scoping exercise. In particular, the School of Optometry and Vision Science at UCC made an ideal platform for investigating gender representation in higher education, as all 11 members of academic staff are male, despite a 33% female undergraduate optometry student cohort. Since 2008, less than 10% of applications for academic posts and internships leading to academic posts have been from women (data taken from UCC’s application records), showing primarily a lack of applications rather than a lack of successful appointments.

Stakeholder workshops were conducted with undergraduate optometry students (Years 4–6; *n* = 199; 54% female) at the two HEIs, using the nominal group technique. Participants were grouped by gender and asked to list their motivations and barriers to pursuing an academic career in vision science. Within each group, responses were discussed, collated and then prioritised by voting to identify (1) the most common and (2) the most critical motivation and barrier. Following compilation across groups, a final voting round identified the motivation and barrier most frequently reported, and those considered most impactful, stratified by gender.

Several key themes emerged from this exploratory work, with reported motivators and barriers spanning academic, financial, social and personal domains. There was significant overlap in themes identified by men and women. Common motivations included a passion for the subject, good remuneration and prestige. Women highlighted the need for career mentorship as critical, while men cited self-improvement and the opportunity to contribute to training the next generation. With respect to barriers, men and women both cited long programme duration and financial constraints. Men emphasised the lack of adequate research facilities at their institutions as critical, while women emphasised the difficulties of combining academia with marriage and family commitments.

These results highlighted both shared and gender-specific career motivators and barriers, offering valuable insights to inform the next stage of the project – establishing a network to support women aspiring to academic careers. This is where the ‘Women in Vision Ghana’ network was born.

Networks designed to support women exist in many disciplines around the world. The scope of these networks ranges from institutional (e.g. EMPOWER Network at Cardiff University^9^), to national (e.g. Women in Healthcare^10^), to global (e.g. Women in Academia Support Network^11^), but they all share a similar remit, supporting women in their respective fields. The Women in Vision UK network, one of the partners on this project, is open to all vision-related disciplines within the United Kingdom, and fosters new collaborations, establishes mentoring connections and raises the profile of women working in vision across the United Kingdom.^12^ Founded in 2017, the network has grown to ~250 paying members, with its influence further extended through a mailing list with over 475 subscribers.

The strength of a network lies in its members; it is therefore crucial to shape the network around their needs and priorities. To this end, Women in Vision UK runs regular membership surveys for members to provide feedback on the network’s effectiveness, goals and future direction. This process ensures the network can continually evolve in response to members’ needs. The 2024 survey highlighted the value of the network in promoting support rather than competition, and the critical role that mentorship plays. Barriers and motivations are very likely to differ between countries, and it was speculated that a locally run vision network in Ghana could better meet the needs of women within these disciplines than a more widely reaching network. Within this project, Women in Vision UK, UCC and Cardiff University supported the newly formed Women in Vision Ghana committee. The collaboration has allowed both Women in Vision committees to share their experiences and ideas, promoting and strengthening both networks.

The Women in Vision Ghana network was formally launched at its inaugural conference in July 2025, at UCC (Figure 1). Hosted by the School of Optometry and Vision Science, UCC, under the deanship of Prof. Stephen Ocansey, the conference was chaired by the Pro-Vice Chancellor of UCC, Prof. Denis Aheto. It was attended in person by more than 250 people and was streamed online for those unable to attend, receiving over 600 views to date. The majority of attendees were undergraduate students in optometry and allied health professions from UCC and KNUST, as well as teaching faculty and qualified eye care practitioners. The programme began with an exhibition for local high school students studying STEM subjects, to promote careers in eye care and academia. Attendance at all events during the day was open to all, irrespective of gender, as it is important to remember the allyship role of men in driving change.

**Figure 1.**
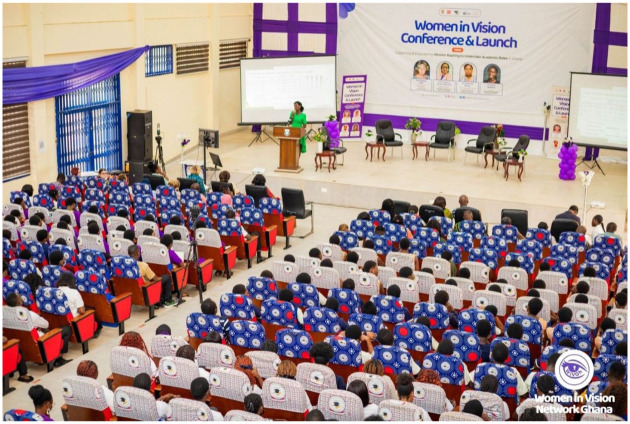
The inaugural conference of the Women in Vision Ghana network was held at the University of Cape Coast, with over 250 attendees.

The main conference session was then introduced with an overview of the project by project lead, Dr Louise Terry from Cardiff University, and co-lead, Dr Enyam Morny from UCC. The theme of the conference was “*Supporting and empowering women aspiring to undertake academic roles in Ghana*”, aligning with the aims of the new network. Dr Dillys Amega, researcher from UCC and Chair of the newly established Women in Vision Ghana network, gave a summary of the findings from the scoping exercise, as outlined above. The keynote on myopia management was delivered by Dr Sylvia Agyekum, and words of support from the sister network, Women in Vision UK, were given by project co-lead Dr Lindsay Rountree.

Prof. Barbara Ryan from Cardiff University spoke about her personal journey in eye care across Sub-Saharan Africa and the UK, urging attendees to remember that a career is a marathon, not a sprint. An interactive panel discussion, comprising female academics, gave attendees (including those joining remotely) an opportunity to ask questions of women established in vision science. This part of the programme received particularly positive feedback, with discussions primarily focused on personal career journeys and work–life balance. The programme was brought to a close with the inauguration of the founding committee members and the formal launch of the Women in Vision Ghana network (accompanied by suitable fanfare) by President of the Ghana Optometric Association, Prof. Samuel Bert Boadi-Kusi and Prof. Angela Amedo Kwarteng, the first Professor of Optometry in Ghana.

Events like this not only increase the visibility of current gender inequities in academia but also amplify the impact of initiatives aimed at addressing them. The reaction to the network launch has been uplifting, with a current membership of over 200 people across Ghana, and several Ghanaians who now live and work abroad. The founding committee is actively organising regular training and networking events to upskill and inspire members, including journal clubs, and establishing a mentorship scheme. A core philosophy of the network is widening participation, and as such, hybrid events are held wherever possible to enable the inclusion of those unable to join an event in person. Sustainability is a key priority, and with ongoing support from Women in Vision UK, the committee is seeking sponsorship from stakeholders to host annual conferences that further support its members.

It is encouraging to see a growing number of organisations aiming to address gender inequities in healthcare leadership and academia, such as the recent ‘Women Leaders in Eye Health’ conference, held in Ghana by non-profit NGO, Orbis International.^13^ While these networks share many core values, their presence should not be seen as overcrowding the space. Rather, their remits are complementary, supporting the advancement and progression of women through both academic and clinical careers. The authors advocate for openness and collaboration between synergistic networks, working together to maximise reach and impact, and drive meaningful progress towards gender equity. To support this, we urge stakeholders across the academic and healthcare sectors to dedicate funding to women-led initiatives, providing opportunities and helping to overcome some of the barriers women face in these spaces.

Despite ongoing progress, women remain underrepresented in healthcare leadership and academia, particularly in Sub-Saharan Africa. The Women in Vision Ghana network is a locally led, grassroots, member-driven initiative, aiming to address these inequities by providing mentorship, training and networking opportunities. Drawing on lessons learned from networks such as Women in Vision UK, the network has rapidly built membership and engagement, and its ongoing impact will be monitored through engagement metrics, academic outputs, and application, appointment and career progression rates by gender. Sustainable and accessible networks like these play a critical role in empowering women to pursue academic and clinical leadership roles, helping to narrow the global gender gap.

## Declarations

**Ethics approval and consent to participate:** Not applicable.

**Consent for publication:** Not applicable.

**Availability of data and materials:** Not applicable.
